# 

**Published:** 1915-04-03

**Authors:** 


### The Distinguished Service Order.

The following is among those whose appointment to be Companions of the Distinguished Service Order for gallantry and devotion to duty while serving with the Expeditionary Force has been approved by the King: Lieutenant Edmund Hume Moore, M.B., Royal Army Medical Corps, attached 2nd Battalion the Leicestershire Regiment.

For conspicuous gallantry on February 23, 1915, near Richebourg L'Avoue, in going out with another officer and a stretcher-bearer to within 150 yards of the enemy and attending to a severely wounded soldier. The stretcher-bearer was then wounded, and Lieutenant Moore remained in attendance on him, undoubtedly saving his life.

On the next day this officer again went with the greatest gallantry to the assistance of a wounded man under the aimed fire of the enemy. He dressed the man's wounds, and was immediately afterwards wounded himself.

RATES OF SUBSCRIPTION: Twelve months, 6/6; Six months, 4/-; Three months, 2/-, post free to any address in the United Kingdom. Abroad, Twelve months, 8/8; Six months, 5/-; Three months, 2/6, post free.—Address The Subscription Dept., "THE HOSPITAL," The Hospital Building, 28/29 Southampton Street, Strand, London, W.C.

